# “You are wasting our drugs”: health service barriers to HIV treatment for sex workers in Zimbabwe

**DOI:** 10.1186/1471-2458-13-698

**Published:** 2013-07-31

**Authors:** Sibongile Mtetwa, Joanna Busza, Samson Chidiya, Stanley Mungofa, Frances Cowan

**Affiliations:** 1 Zimbabwe AIDS Prevention Project, Department of Community Medicine, University of Zimbabwe College of Health Sciences, Harare, Zimbabwe; 2 Centre for Sexual Health, HIV and AIDS Research Zimbabwe, 21 Rowland Square, Harare, Zimbabwe; 3 Department of Population Health, London School of Hygiene and Tropical Medicine, London, UK; 4 UNFPA, Harare, Zimbabwe; 5 City of Harare Health Department, Harare, Zimbabwe; 6 Research Department of Infection & Population Health, University College London, Centre for Sexual Health & HIV Research, London, United Kingdom

## Abstract

**Background:**

Although disproportionately affected by HIV, sex workers (SWs) remain neglected by efforts to expand access to antiretroviral treatment (ART). In Zimbabwe, despite the existence of well-attended services targeted to female SWs, fewer than half of women diagnosed with HIV took up referrals for assessment and ART initiation; just 14% attended more than one appointment. We conducted a qualitative study to explore the reasons for non-attendance and the high rate of attrition.

**Methods:**

Three focus group discussions (FGD) were conducted in Harare with HIV-positive SWs referred from the ‘Sisters with a Voice’ programme to a public HIV clinic for ART eligibility screening and enrolment. Focus groups explored SWs’ experiences and perceptions of seeking care, with a focus on how managing HIV interacted with challenges specific to being a sex worker. FGD transcripts were analyzed by identifying emerging and recurring themes that were specifically related to interactions with health services and how these affected decision-making around HIV treatment uptake and retention in care.

**Results:**

SWs emphasised supply-side barriers, such as being demeaned and humiliated by health workers, reflecting broader social stigma surrounding their work. Sex workers were particularly sensitive to being identified and belittled within the health care environment. Demand-side barriers also featured, including competing time commitments and costs of transport and some treatment, reflecting SWs’ marginalised socio-economic position.

**Conclusion:**

Improving treatment access for SWs is critical for their own health, programme equity, and public health benefit. Programmes working to reduce SW attrition from HIV care need to proactively address the quality and environment of public services. Sensitising health workers through specialised training, refining referral systems from sex-worker friendly clinics into the national system, and providing opportunities for SW to collectively organise for improved treatment and rights might help alleviate the barriers to treatment initiation and attention currently faced by SW.

## Background

Globally, sex workers are disproportionately affected by HIV. A recent systematic review found that HIV prevalence among sex workers ranges between 10–18 times higher than that of the general population of women of reproductive age [1]. The 2008 study they cite for Zimbabwe reported 61.2% prevalence among 214 sex workers [2], while in 2002 the Zimbabwe National AIDS Council (NAC) reported 64-75% HIV prevalence among sex workers and women living near plantations and mining areas [3]. The most recent country report to UNAIDS gives an estimate of 50% [4]. These figures are consistent with HIV prevalence data on sex worker populations throughout the region, for example, 59.6% in South Africa, 70.7% in Malawi, and 45.1% in Kenya [1].

Sex workers’ share of the HIV burden has not been matched by commensurate efforts to provide access to antiretroviral treatment (ART) [5], despite calls as early as 2002 for targeting treatment programmes to female sex workers as a means to slow national epidemics [6]. These largely went unheeded until evidence emerged of sex work’s role in driving HIV even in generalised epidemics [7] and the potential contribution of providing ART to sex workers in the form of “treatment as prevention” [8,9]. At the same time, the “Universal Access” agenda has made equity a guiding principle for national programmes, leading to greater prioritisation of key populations, including sex workers [10,11]. Arguments for social justice have converged with pragmatic imperatives, leading to reinvigorated efforts to reach sex workers with HIV testing and treatment [12].

### HIV treatment for sex workers

The extent to which sex workers link to and are retained in HIV care as well as achieve adequate levels of adherence will determine both coverage equity and epidemiological impact. To date, there is scant literature on treatment tailored to sex workers, and little reported on their retention across the treatment cascade. One study from Burkina Faso found that while sex workers reported adherence that did not differ significantly from that of other women enrolled in ART, they had poorer virological outcomes and higher rates of treatment failure within the first six months (20.6% vs. 2.8% p = 0.03) [13]. In Benin, 371 ART patients were followed for a year, and the 53 sex workers in the sample had lower CD4 gains, higher detectable viral loads, and a higher mortality rate than others in the cohort, attributed to lower reported adherence [14]. Qualitative research conducted in Kenya during a safety and acceptability trial of pre-exposure prophylaxis (PrEP) found high rates of motivation and commitment to PrEP among female sex workers, but also numerous challenges to adherence related to frequent mobility, disruption by alcohol use, inconvenience of post-coital dosing requirements, and concerns about inadvertent disclosure of engagement in sex work [15]. Similarly, findings from a study in Chennai, India identified a wide range of inter-related barriers confronted by female sex workers in accessing treatment, such as unsupportive family members, discrimination against both sex workers and people living with HIV, and service-related factors including poor quality care and stigmatization from health staff [16].

These studies demonstrate that, for the most part, sex workers experience the same barriers as the general population to accessing HIV treatment reported throughout the literature on treatment programmes in resource poor settings [17]. Key barriers identified in a wide range of settings include direct and opportunity costs of attending services [18]; distance to facilities and poor or expensive transport [19]; lack of support from family or others, particularly if HIV status has not been disclosed [20]; dissatisfaction with the quality or delivery of services [21,22]; competing health or religious beliefs [23,24]; and anxiety induced by pervasive HIV-related social stigma [25].

The challenges faced by sex workers are further exacerbated by the additional stigma attached to their work [26]. As ART programmes are scaled-up, understanding how sex workers navigate barriers to service use will be critical for developing feasible interventions to support their engagement in care and subsequent treatment adherence and to monitoring national responses to HIV [27].

### Study design

We conducted a qualitative study to examine determinants of female sex workers’ attrition in the early stages of a programme designed to improve uptake of testing and access to ART among sex workers in Harare. We conducted this study within the context of Zimbabwe’s national HIV prevention programme for sex workers, which includes provision of fixed-site and mobile clinical services for sex workers and a peer education component. After reviewing routine service statistics, we designed a qualitative study to explore HIV positive sex workers’ experience of barriers and facilitators to engagement with care, with a view to identifying measures that could be used within our programme to develop feasible interventions supporting sustained retention.

### Study setting

Despite the criminalisation of sex work, the Zimbabwe National AIDS Strategic Plan for 2006–2010 (ZNASP I) identified sex workers as a key population that was particularly vulnerable to HIV. This led the National AIDS Council to conduct a situational analysis with its partners, which recommended introduction of targeted interventions for sex workers as part of the national response to HIV. In 2009, Sisters with a Voice (SWV) was established under the auspices of the National AIDS Council (NAC), funded by UNFPA, and implemented through the Zimbabwe AIDS Prevention Programme-University of Zimbabwe.

The overall aims of SWV are to i) reduce HIV acquisition among sex workers; ii) reduce HIV transmission to their clients; and iii) improve sex workers’ rights, through providing clinical services supported by peer educators and community outreach. Initially one static and four mobile sex worker health centres were established; services are provided free of charge and included health education, syndromic management of sexually transmitted infections (STI), condom distribution, contraception, provider initiated HIV testing and counselling (PITC), referral for CD4 counts and ART initiation, and referrals to organisations providing legal advice.

A review of routine medical records in 2010 revealed that while numerous sex workers received HIV testing and STI treatment, few took up referrals to HIV treatment services, which remained part of the national public health system. Assisted referrals system was established, which support sex workers’ registration with ART services. Programme staff physically accompanied women who tested HIV positive to the ART clinic and paid for their registration and CD4 count directly. However, despite this targeted assistance, of the 136 HIV positive sex workers who were supported to attend their first referral appointment, only 66 (48.5%) subsequently attended ART services at least once, with just 20 (14.7%) attending follow up visits. We designed this qualitative study to explore why women were not retained in care following assisted referral from the sex work programme.

## Methods

We held three Focus Group Discussions (FGDs) between January and March 2011 with 38 women who had been referred from the SWV clinic for management of HIV. A complete list of referrals was generated from SWV records and organised by date of clinic visit; every 3^rd^ woman referred was invited to participate in FGDs. The programme outreach worker contacted selected women, described the study, and invited them to participate; all agreed and were randomly assigned to one of three FGD of 12–14 participants. Focus groups were selected in order to provide group camaderie while sensitive issues were raised and to encourage women to share experiences, build each other’s confidence, and prompt group insights. Prior programme experience suggested that sex workers would feel individual interviews might “single them out”. We also expected interviews to be more susceptible to desirability bias, given the role SWV had played in facilitating SW’ access to care. SW knew that all FGD participants were HIV-positive and attending a discussion would enable their status to be known by others. Ethical approval for the study was obtained from the Medical Research Council of Zimbabwe. All participants provided written informed consent and were given a copy of their own signed consent to keep. The consent form specified that living with HIV was a selection criteria and thus participants’ HIV status would be disclosed to other members participating in that FGD.

Discussions were conducted in Shona by the first author, and explored sex workers’ experiences and perceptions of health services, particularly at the HIV treatment facility to which they had been referred. Questions elicited accounts of health-seeking behaviour, and focused on how managing HIV interacted with challenges specific to being a sex worker.

FGDs were digitally recorded, transcribed in Shona, and translated into English. The transcripts were analysed following familiarisation of the data, and recurring themes identified based on both categorisation of barriers obtained from the literature, as well as emerging topic areas and ideas specific to this study context. We present findings divided into *supply side* barriers, which sex workers described as related to health service delivery, and *demand side* barriers, identified as factors that hindered their ability to seek or maintain care. For both, we highlight sex workers’ understanding of how their involvement in sex work directly shapes their experience of HIV treatment.

## Results

### Background characteristics of participating sex workers

Among the 38 participants, 10 were single (never married), 14 were divorced and 14 widowed. Five were aged 18–25 years, 22 were aged between 26–33 years, 8 aged between 34–41 years and 3 were between 42–48 years. The majority of them had gone to school up to primary school level (26 primary school, 11 secondary and 2 tertiary). Although reasons for practicing sex work were not explored in this study, the fact that most were widowed or divorced suggests sex work may have been a strategy to deal with financial constraints. Women worked in a range of settings, including night clubs, bars, on the street and at home. All participants had been referred for ART care to a centrally located public clinic in Harare that does not provide special services adapted to sex workers.

### Supply side barriers

Participants emphasised service-related determinants of attrition, regardless of their level of interaction with the hospital to which they had been referred. Whether women had persevered in pursuing HIV treatment, dropped out early, or never attended their first appointment, reports of active discrimination from hospital staff dominated their narratives. Women with direct experience of the clinic described how hospital nurses openly expressed their hostility to sex workers, and conducted examinations and counselling with a negative attitude:

She opened my file and I saw her face just changed instantly, and she actually frowned and looked at me like I was disgusting her. Her first words to me were, ‘so you are a prostitute and you actually have the guts to come here to waste our time and drugs on you, why do you do such things anyway? Why can’t you find a man of your own and get married’? (SWH006, FGD2, 32 years old)

The nurse said to me, ‘how can you, a sex worker, even have high blood pressure? It’s high because of too much sex…you are wasting our drugs instead of us giving them to those who have proper high blood pressure, caused by women like you when you take and infect their husbands’ (SWH032, FGD3, 39 years old)

Public humiliation was considered an integral part of treatment for sex workers, and most women who had gone to the referral hospital felt they would not access services there again. In all 3 FGDs, women described how hospital staff would to the waiting area and make public announcements that all the sex workers present should go queue at the back or stand in a separate line:

We were in the queue with everyone else when suddenly one of the nurses came out and loudly said ’the sex workers who have come … please go and queue at the back of this line, we will attend to you last’. Everyone there turned and you could see they were all eager to see who these women were. We dragged our feet and went to the back. Luckily there were six of us, so at least the embarrassment and humiliation was somehow shared amongst ourselves and we just had to pretend like we didn’t care. I remember one lady who had also been referred from here actually walked away and left, we never saw her again… shame/ embarrassment is worse than death, ladies! (SWH003, FGD1, 29 years old)

Occasionally staff appeared accompanied by pastors from a local church, who came to preach at the hospital, and also publicly humiliated sex workers as they waited for their appointments. One woman reported meeting a pastor who felt sex workers needed some form of “cleansing”, and proceeded to “pray” for them.

*“…all the prostitutes that are being mentioned here … come forward*, *come and stand in this corner right now so that we can lay hands on you and pray for you*!*” There were six of us there and we were dragged into a corner and they started praying with their hands on our heads and speaking in tongues. It was terrible and I was now crying as this woman kept shaking my head saying ‘demons of prostitution-come out in the name of Jesus…’ (SWH001, FGD1, 30 years old)*

Word had clearly spread among sex workers of such demeaning episodes, deterring some from taking up their initial referral. Fear of being mistreated actively dissuaded at least three women from ever attending:

It’s just the thought of being seen as a sex worker that gives me the shivers to go there, I am scared that they will shout or humiliate me, as I heard they are good at doing that… (SWH013,FGD2, 20 years old)

Concerns about being identified as a sex worker were exacerbated by use of referral forms provided at the SWV clinic. Several respondents felt that these cards prompted insensitive comments from nurses who recognised the referral cards as originating from the sex worker service. When woman presented their referral letter, nurses would start frowning and ridiculing them reinforcing women's reluctance to take up referrals. One woman quoted her friend who refused to go to the hospital through the free channel, saying she preferred to wait until she could afford to pay herself:

*This letter sells me out at that clinic, then those nurses will humiliate me in front of everyone. It’s for free with this letter, but I will not go…I would rather stay at home until I get my own money to go there…the card is like a tattoo written, “sex worker! Bewar*e!” (SWH033,FGD3, 40 years old)

Participants discussed feeling embarrassed, shy, and unworthy of the service because of the stigma associated with their work. Internalised shame and anxiety about being known to be a sex worker reduced women’s confidence to attend treatment services:

It’s embarrassing, you know, to be seen as a sex worker by the health workers, especially if you come from the same area. If it is me I will just walk away and never come back… (SWH028,FGD3, 35 years old)

While criticism centred on the referral hospital’s atmosphere of discrimination and disrespect, sex workers also reported a range of service-related problems that reflect wider issues of Zimbabwe’s health system. For example, participants complained that staff had no sense of urgency when doing their work. They expressed dismay over the staff’s strict observation of tea and lunch times, during which waiting patients were left unattended:

It was as if they were dragging [their feet], just waiting to go off for tea and lunch time. I was hungry myself and I felt like leaving to go home to eat, then come back the next day. Then l realized that this will be the same situation the next day, so I just stayed, but I’m sure some people who are not strong enough cannot hold on the whole day, they will leave and simply not come back (SWH015,FGD2,32 years old)

Sex workers reported they would spend up to 8 hours at the hospital, wasting the whole day instead of doing something productive to earn money. Some work as vegetable vendors during the day so attending the hospital resulted in loss of income. Thus they preferred to wait until their condition seriously deteriorated before attending the hospital:

*It’s better to go there when you are really sick because they waste our time… (SWH008,FGD1, 48 years old*)

### Demand side barriers

Participants in our study mentioned a range of other factors that limited their retention in care. Financial and logistical barriers impeded health service use. For instance, although the SWV programme paid the initial consultation fee and for CD4 count test, other charges are associated with treatment, women had to bear other costs such a US$ 10 fee for consulting a doctor for management of opportunistic infections. As women usually earned $5 for a “short time quickie” (sex with a client for not more than 20 minutes), or just $1 when desperate, such out-of-pocket medical costs were prohibitively expensive.

Respondents also worried that ART patients require more nutritious diets than a “normal” person, necessitating changes in their routine feeding habits. They felt that ARV drugs “were too strong” to take along with their regular diet of “sadza” (ground corn) and “muriwo” (green vegetables). One woman mentioned that taking ARVs made her eat more food and this was seen as an expense:

Have you also noticed that when you are on ARVs you eat a lot? You feel hungry all the time…with the little food some of us have, you can’t afford to be eating like that so you end up tempted to stop going there to get them. (SWH015,FGD2, 32 years old)

Travelling time was also perceived as a barrier to treatment. Some sex workers reported having to travel the day before their appointment so as to arrive as soon as the clinic opened. This was seen as tiring, boring and wasting productive time when they could be earning.

## Discussion

Despite renewed interest in extending HIV treatment to sex worker communities, this study confirms that a wide range of health systems and structural barriers can contribute to high attrition rates, even when targeted and “sex worker friendly” services exist. Under 15% of HIV-positive sex workers who were referred from the SWV clinics for free specialised care attended more than one scheduled appointment, and over half did not take up referral at all. The sex workers recruited into this study were those who had already successfully attended a targeted prevention programme and undergone HIV testing, suggesting there are likely to be many others who have not even entered these early stages of the care continuum.

Supply-side barriers dominated participants’ descriptions of the challenges they faced in seeking treatment, particularly the openly hostile attitudes and degrading behaviour of health staff. Sex workers were keenly aware of the stigma they faced. They reported being dissuaded from engaging in care by the stories they heard from their peers of the public humiliation and disrespect they could expect at the hospital. Discrimination from health care workers has been found to impede health-seeking behaviour of sex workers in a wide range of contexts [28-30], and dissatisfaction with waiting times or quality of care has been shown to cause sex workers to attend private or informal services even when free treatment is available [31,32].

Demand-side barriers also played a role, and while these are shared by many people in HIV treatment, sex workers may be particularly vulnerable to them not only because of their socioeconomic position, but because their marginalised status in society makes them less able to draw on the kinds of social and material support available to other community members [33,34]. Furthermore, sex workers’ exacerbated experience of supply-side barriers may make efforts to overcome other barriers seem futile.

There is a wealth of literature highlighting how criminalisation and other forms of discrimination shape sex workers’ vulnerability [35,36]. Calls for legal reform and structural change have been used to argue for improvement in sex workers’ human rights, including equitable access to the health and social services they need [37].

While our findings reaffirm the need for large scale shifts in social attitudes in Zimbabwe, our more immediate interests lie in how the SWV programme can address the barriers identified in this study and bring about rapid improvement in sex workers’ uptake and retention in HIV treatment. Over the past year, the programme has initiated several measures to reduce the barriers, stigma and discrimination confronted by sex workers in all clinic sites. First, we developed a hands-on training programme for nurses in the public sector. The training directly addressed nurses’ negative attitudes towards sex workers, including through the use of a video produced and acted in by sex workers from the SWV programme. Narratives in the video described circumstances that had led SW into selling sex, and described how some learned of they had contracted HIV during marriage rather than through their work. Following this sensitisation, nurses were brought into SWV clinics for short practice-based modules in sex worker “friendly” service provision. They shadowed the SWV clinical staff, but more importantly, attended sessions run by sex workers who described their experiences with discrimination within the health system. Regular rotations are planned to allow a continuous stream of nurses from public ART services to cycle through SWV clinics, and monthly feedback meetings are held with those already trained so they continue to share their experiences treating sex workers, and refresh commitment to greater tolerance.

Second, the peer education programme has been expanded into a more inclusive programme of community mobilisation, based on successful models from other settings [38-41]. A total of 20 community mobilisation meetings have been held with over 200 sex workers to discuss priorities, build group solidarity, and empower them to seek early treatment for HIV and other diseases and demand their right to good quality services and humane treatment. In addition to building sex workers’ confidence in challenging the behaviour of health staff, participatory groups may help sex workers overcome some of the demand-side barriers they face as well, by developing peer networks and mutual trust, and thus increasing their access to both material and psychosocial support [34,42].

These are new initiatives, and we hope to continue to strengthen the SWV programme and advocate for improvements in the public sector. However, we also note that the SWV programme itself has the potential to identify SW as “different” to other clients at the ART centres. Efforts to make registration and attendance affordable and less daunting appear to have inadvertently contributed to some SW’s perception that they can be identified and thus will receive further discrimination. Addressing this may require intensified efforts to change attitudes among public providers and make referral forms and the overall referral process more discreet. Another option, however, is to decentralise HIV care and provide specialised “sex worker friendly” clinics to operate. We plan to reach new sites with SWV and introduce ART provision within the programme to avoid the need for referrals where attrition remains high. While there is clearly a need for more sex worker-specific clinics to improve coverage, in the long term, sex workers’ treatment needs to be integrated into the national response to ensure programme equity and sustainability.

### Limitations

Due to financial constraints, the study was limited to one SWV site, Harare. A larger study conducted in other sites across the country may have identified different barriers or priorities. Demand-side barriers may have been more important in rural locations where distances to facilities are greater and social acceptance of sex workers even lower. Furthermore, as mentioned previously, our study participants were women, who the researcher knew were benefiting from targeted services, were aware of their status, had considered initiating ART, and were willing to discuss these issues. They are unlikely to represent the wider population of HIV-positive sex workers, who might face even greater challenges and barriers.

## Conclusion

Improving access to ART for sex workers is critical for their own health, for equity in national programmes, and for public health benefit. Targeted interventions may not be adequate to ensure sex workers remain engaged across the care continuum and achieve adherence levels required for treatment success. Great attention needs to be paid to links within the health system, and how sex worker friendly services can work to reduce discrimination faced by sex workers outside their own clinics and programmes. Referral mechanisms need to be monitored, and both supply and demand side barriers addressed through existing programmes. Interventions can include training public health care providers, mobilising sex workers around treatment literacy and psycho-social support, decentralising ART, changing the social mores and ultimately working towards the decriminalization of sex work.

## Competing interests

The authors declare that they have no competing interests.

## Authors’ contributions

SM conceived of the study, participated in its design and coordination,interviwed participants and drafted the manuscript. JB participated in the study design, helped to draft and participated in the final writing of the manuscript. SD helped in drafting the final manuscript. SM helped in drafting the final manuscript. FC provided technical support participated in the study design and helped in the drafting and final write up of the manuscript. All authors read and approved the final manuscript.

## Pre-publication history

The pre-publication history for this paper can be accessed here:

http://www.biomedcentral.com/1471-2458/13/698/prepub
